# Books and Magazines

**Published:** 1909-12

**Authors:** 


### Books and Magazines.

DIAGNOSIS. By J. C. Wilson, A. M., M. D., Professor of the Practice of Medicine and Clinical Medicine in the Jefferson Medical College, and Physician to its Hospital; Physician to the Pennsylvania Hospital; Physician in Chief to the German Hospital, Philadelphia. Octavo; 1478 pages; 300 illustrations; cloth, $6.00. J. B. Lippincott Co., Philadelphia, since 1792. London, since 1872. Montreal, since 1897.

This volume has been written to meet the urgent demands of many successive classes of pupils, and in the hope that a simple and direct presentation of the subject of medical diagnosis will prove useful to the profession at large.

The treatment of the subject-matter under four main headings has been adopted, with a view of simplifying the arrangement of the topics in a department of medicine which has attained large scope and insistent importance. This plan will fulfill the twofold requirement, that, within the compass of a single book, clinical phenomena on the one hand, and on the other those complexes of clinical phenomena which constitute diseases, are brought into correlation in such a manner that the practitioner who seeks information upon an obscure case may at once turn to the discussion of the methods available to clear it up, and the student may find the definite clinical applications of the same methods and their results in Descriptive Medicine.

Practical rather than theoretical considerations have been held constantly in view alike in the treatment of the clinical and the laboratory subjects. To attain this end a degree of positiveness of assertion not warranted under other circumstances and the avoidance of the discussion of moot and unsettled questions have seemed proper.

To add to the list of works on diagnosis demands the justification of something different in method, new arrangement of detail, and the presentation of the whole subject in accordance with the requirements of contemporary medicine. In the present volume these demands are fulfilled. It is the outcome of many years devoted to work in the wards with the controlling side-lights upon bedside diagnosis afforded by the clinical laboratory, revelations at the hands of surgical colleagues in the operating theater and confrères in pathology in the post-mortem room, the frequent opportunity of seeing unusual and grave cases in consultation, and long experience as a teacher. Such a career arouses enthusiasm but begets caution.

The illustrations are in a large part drawn from personal observations. They have been selected solely with the view to elucidate the subject in hand. Diagrams have been employed when this method of presentation has appeared desirable, and the free use of clinical charts constitutes an important feature of the work.

